# The apolipoprotein A-I mimetic peptide, ETC-642, reduces chronic vascular inflammation in the rabbit

**DOI:** 10.1186/1476-511X-10-224

**Published:** 2011-11-30

**Authors:** Belinda A Di Bartolo, Laura Z Vanags, Joanne TM Tan, Shisan Bao, Kerry-Anne Rye, Philip J Barter, Christina A Bursill

**Affiliations:** 1 Lipid Research Group, Heart Research Institute, 7 Eliza St, Newtown, NSW, 2042, Australia; 2 Immunobiology Unit, Heart Research Institute, 7 Eliza St, Newtown, NSW, 2042, Australia; 3 Discipline of Pathology, University of Sydney, Camperdown, NSW, 2050, Australia; 4 Department of Medicine, University of Sydney, Camperdown, NSW, 2050, Australia; 5 Department of Medicine, University of Melbourne, Parkville, Victoria, 3010, Australia

**Keywords:** High-density lipoproteins, apolipoproteinA-I, apolipoproteinA-I mimetic peptides, vascular inflammation, rabbits, intracellular cell adhesion molecule-1 (ICAM-1) and vascular cell adhesion molecule-1 (VCAM-1)

## Abstract

**Background:**

High-density lipoproteins (HDL) and their main apolipoprotein, apoA-I, exhibit anti-inflammatory properties. The development of peptides that mimic HDL apolipoproteins offers a promising strategy to reduce inflammatory disease. This study aimed to compare the anti-inflammatory effects of ETC-642, an apoA-I mimetic peptide, with that of discoidal reconstituted HDL (rHDL), consisting of full-length apoA-I complexed with phosphatidylcholine, in rabbits with chronic vascular inflammation.

**Results:**

New Zealand White rabbits (n = 10/group) were placed on chow supplemented with 0.2% (w/w) cholesterol for 6-weeks. The animals received two infusions of saline, rHDL (8 mg/kg apoA-I) or ETC-642 (30 mg/kg peptide) on the third and fifth days of the final week. The infusions of rHDL and ETC-642 were able to significantly reduce cholesterol-induced expression of intracellular cell adhesion molecule-1 (ICAM-1) and vascular cell adhesion molecule-1 (VCAM-1) in the thoracic aorta (p < 0.05). When isolated rabbit HDL was pre-incubated with human coronary artery endothelial cells (HCAECs), prior to stimulation with TNF-α, it was found that HDL from ETC-642 treated rabbits were more effective at inhibiting the TNF-α-induced increase in ICAM-1, VCAM-1 and p65 than HDL isolated from saline treated rabbits (p < 0.05). There were, however, no changes in HDL lipid composition between treatment groups.

**Conclusions:**

Infusion of ETC-642 causes anti-inflammatory effects that are comparable to rHDL in an animal model of chronic vascular inflammation and highlights that apoA-I mimetic peptides present a viable strategy for the treatment of inflammatory disease.

## Background

An increase in the endothelial cell expression of adhesion molecules such as vascular cell adhesion molecule-1 (VCAM-1) and intercellular adhesion molecule-1 (ICAM-1) is characteristic of the initial inflammatory response triggered by endothelial damage or dysfunction [1]. Elevated expression of adhesion molecules promotes the recruitment and trans-endothelial migration of circulating monocytes into the artery wall, eventually leading to the development of atherosclerosis [1].

The anti-inflammatory properties of high-density lipoproteins (HDL) are well established [2]. *In vitro *studies have demonstrated that reconstituted (rHDL), containing apolipoprotein (apo) A-I (the main apolipoprotein constituent of HDL) complexed with phospholipids, inhibit the expression of VCAM-1 and ICAM-1 in human umbilical vein endothelial cells [3-6]. Consistent with this, *in vivo *studies in rabbits also show that lipid free apoA-I and rHDL reduce the expression of arterial VCAM-1 and ICAM-1 in the peri-arterial cuff model of acute inflammation [3,7,8]. Due to their potent anti-inflammatory properties, both HDL and apoA-I have immense therapeutic potential, but despite this there is currently no translated use to clinic.

The limiting factor in the therapeutic usefulness of apoA-I is its relatively large size of 243 amino acids, thereby making its synthesis difficult. This has lead to the development of apoA-I mimetic peptides that are much shorter in length (18-22 peptides) and able to be readily synthesized on a large scale, but still exhibit the same beneficial properties as HDL and full-length apoA-I. For example, infusions of mimetic peptides reduce atherosclerotic lesion size, improve endothelial dysfunction and also inhibit VCAM-1 and ICAM-1 expression *in vitro *and *in vivo *[9-13]. The apoA-I mimetic peptide used in our study, ETC-642, consists of a 22-amino acid synthetic amphipathic peptide complexed with sphingomyelin and 1,2-dipalmitoyl-*sn*-glycero-3-phosphocholine (DPPC) [14,15]. Recent studies have found that ETC-642 is as effective as rHDL at suppressing acute inflammation in the rabbit peri-arterial collar model [16]. The anti-inflammatory effects of ETC-642 on chronic inflammation are, however, currently unknown.

Accordingly, this study has investigated the effect of ETC-642 on low-grade chronic vascular inflammation, in cholesterol-fed New Zealand White (NZW) rabbits [17,18]. We find that ETC-642 reduced the expression of VCAM-1 and ICAM-1 in the rabbit thoracic aorta to a similar extent as rHDL containing full-length apoA-I. These studies highlight the efficacy and therapeutic potential of mimetic peptides in the treatment of inflammation and cardiovascular disease.

## Results

### Effects of the dietary intervention on plasma lipids

The concentrations of plasma total cholesterol, HDL cholesterol and non-HDL are presented in Table 1. Consumption of a chow diet supplemented with 0.2% cholesterol for 6 weeks significantly increased total cholesterol concentrations (~3 fold) in all treatment groups (p < 0.01). At sacrifice, there were no differences in plasma total cholesterol between the control animals and those receiving infusions of rHDL or ETC-642.

**Table 1:** Effects of saline, rHDL and ETC-642 infusions on plasma lipid levels in NZW rabbits

	BASELINE			TIME OF SACRIFICE		
	
	TC (mM/L)	HDL-C (mM/L)	Non HDL-C (mM/L)	TC (mM/L)	HDL-C (mM/L)	Non HDL-C (mM/L)
**SALINE**	0.86 ± 0.07	0.77 ± 0.09	0.09 ± 0.07	2.64 ± 0.29*	0.65 ± 0.03	1.99 ± 0.28*
**rHDL**	0.84 ± 0.06	0.63 ± 0.06	0.21 ± 0.06	2.53 ± 0.49*	0.66 ± 0.06	1.87 ± 0.46*
**ETC-642**	0.99 ± 0.05	0.73 ± 0.04	0.26 ± 0.06	2.87 ± 0.27*	0.61 ± 0.05	2.25 ± 0.26*

NZW rabbits (n = 10/group) were maintained on a diet of chow supplemented with 0.2% (w/w) cholesterol for 6 weeks On days 3 and 5 of the last week of dietary supplementation the animals received two infusions of saline, rHDL (8 mg/kg apoA-I) or ETC-642 (30 mg/kg peptide). Blood samples were collected at baseline and at sacrifice and plasma lipid levels were measured as described in "Materials and Methods". Results are expressed as mean ± SEM. * p < 0.05 compared to baseline.

HDL cholesterol levels were not altered after six weeks of cholesterol feeding. Treatment with rHDL or ETC-642 did not change HDL cholesterol levels, compared to saline infused rabbits, at the time of sacrifice.

Non-HDL cholesterol levels were significantly increased (p < 0.05) with cholesterol feeding in rabbits injected with saline (22-fold), rHDL (9-fold) or ETC-642 (8.5 fold) (p < 0.05 for all). However, at the time of sacrifice there were no significant differences in non-HDL cholesterol concentrations between treatment groups.

#### Effect of rHDL and ETC-642 on chronic vascular inflammation

**Figure 1:**
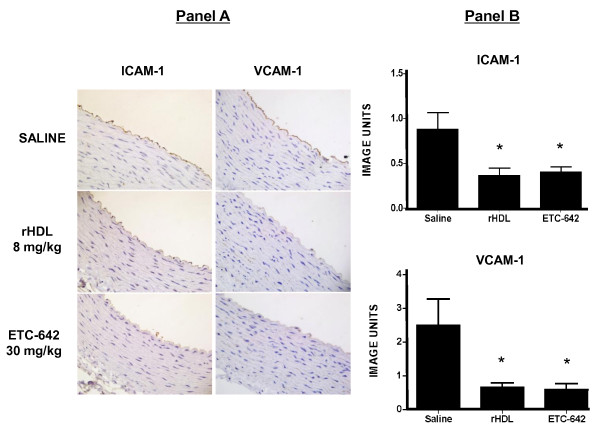
**ETC-642 reduces endothelial adhesion molecule expression**. Panel A: Immunohistochemical staining for ICAM-1 and VCAM-1 in representative thoracic aortic sections from cholesterol-fed NZW rabbits (n = 10 animals/group) infused with either saline, rHDL (8 mg/kg apoA-I) or ETC-642 (30 mg/kg peptide). Panel B: Endothelial expression of ICAM-1 and VCAM-1 were quantified as described in "Materials and Methods". Results are expressed as mean ± SEM. *p < 0.05 and **p < 0.01, compared to saline-infused animals.

The ability of rHDL and ETC-642 to reduce cholesterol-induced inflammation in the thoracic aorta was determined by measuring endothelial ICAM-1 and VCAM-1 expression (Figure 1). After six weeks of cholesterol feeding endothelial ICAM-1 expression was at readily detectable levels in rabbits infused with saline. In rabbits infused with rHDL, ICAM-1 expression was reduced by 58% (p < 0.05). The ETC-642 infusions were as effective as rHDL and reduced endothelial ICAM-1 expression by 53% (p < 0.05). Endothelial VCAM-1 expression was increased to detectable levels following 6 weeks of cholesterol feeding. Two infusions of rHDL or ETC-642 in the final week of cholesterol feeding were found to reduce VCAM-1 by 73% and 76% respectively (p < 0.05).

After six weeks of cholesterol feeding there were no signs of fatty streak formation and therefore macrophages were not detected in the immuno-histochemically stained thoracic aortic sections.

### Effect of ETC-642 on the anti-inflammatory properties and composition of rabbit HDL

**Figure 2:**
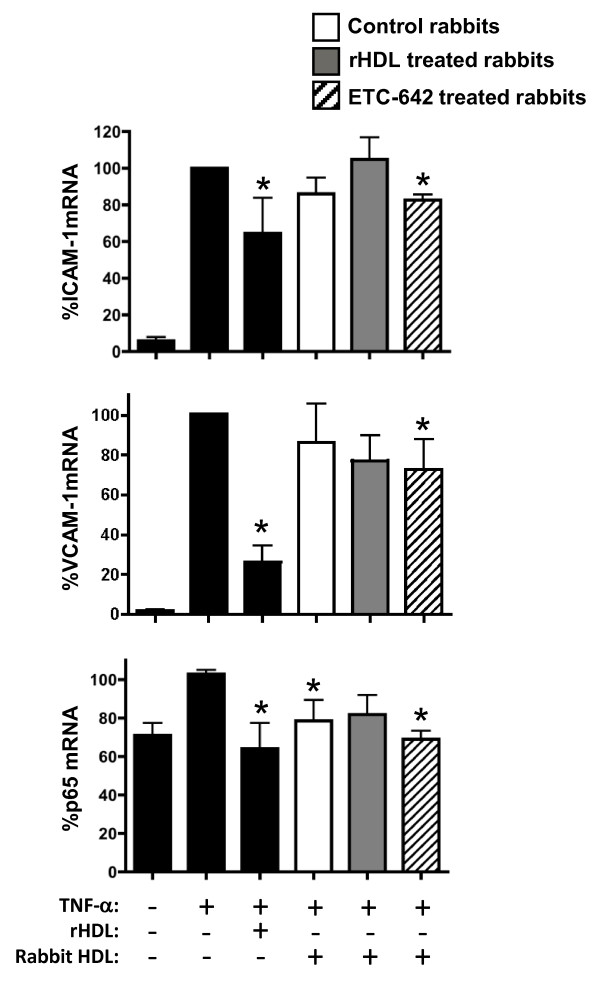
**The effect of isolated rabbit HDL on adhesion molecule expression and NF-κB activation in HCAECs**. HCAECs were pre-incubated for 24 hrs with rHDL or HDL isolated from the plasma of rabbits injected with either saline, rHDL or ETC-642 (n = 10), then stimulated with TNF-α (1 ng/ml) for 5 hrs. ICAM-1, VCAM-1 and p65 mRNA levels were quantified as described in "Materials and Methods". Results expressed as mean ± SEM from triplicate *in vitro *experiments. *p < 0.05 and **p < 0.01, compared to TNF-α stimulated control cells.

HDL were isolated from the rabbits that were infused with saline, rHDL and ETC-642 and assessed for their ability to inhibit TNF-α-induced expression of ICAM-1 and VCAM-1 in HCAECs (Figure 2). As expected, stimulation of HCAECs with TNF-α increased ICAM-1 (17.2 fold) and VCAM-1 (50.6 fold) mRNA levels, compared to un-stimulated control cells (p < 0.001). When the cells were pre-incubated for 24 hrs with rHDL, the TNF-α-mediated increase in ICAM-1 and VCAM-1 mRNA levels was reduced by 35.5 ± 19.4% (p < 0.05) and 73.8 ± 8.4% (p < 0.001), respectively. We found that pre-incubation of cells with HDL from ETC-642 injected rabbits, caused reductions in TNF-α-induced ICAM-1 (17.4 ± 3.0%) and VCAM-1 (27.1 ± 15.0%) mRNA levels (p < 0.05). HCAECs incubated with HDL isolated from rabbits injected with saline and rHDL also expressed lower levels of ICAM-1 and VCAM-1 mRNA, compared to TNF-α stimulated controls, but these reductions did not reach statistical significance.

Changes in the mRNA levels of p65, the active subunit of NF-κB, were also measured, as it is a known regulator of ICAM-1 and VCAM-1 expression. Stimulation of HCAECs with TNF-α increased p65 mRNA levels (1.5 fold), compared to un-stimulated control cells (p < 0.01). When cells were pre-incubated for 24 hrs with rHDL, the TNF-α-mediated increase in p65 mRNA levels was reduced by 36.0 ± 13.4% (p < 0.05). We found that pre-incubation of cells with HDL from rabbits injected with saline and ETC-642 caused reduced TNF-α-induced p65 mRNA levels, by 21.4 ± 10.8% and 31.0 ± 4.3% respectively (p < 0.05). Pre-incubation with HDL from the rabbits that received rHDL did not inhibit p65 mRNA levels.

Infusion of rHDL or ETC-642 did not significantly affect the composition of HDL. The relative concentrations of phospholipid, unesterified cholesterol, cholesterol ester and triglyceride were not statistically different from what was observed for HDL isolated from rabbits infused with saline (Table 2).

**Table 2:** Effects of saline, rHDL and ETC-642 infusions on HDL composition

Infusion	Stoichiometry PL^a^/UC/CE/TG/Protein *(mol/mol)*
**Saline**	27.0 ± 6.8/10.5 ± 1.0/24.0 ± 1.3/1.3 ± 0.2/1.0
**rHDL**	33.5 ± 6.6/7.9 ± 0.9/21.8 ± 1.1/1.8 ± 0.4/1.0
**ETC-642**	32.5 ± 10.2/10.8 ± 1.0/22.1 ± 2.0/1.4 ± 0.3/1.0

NZW rabbits (n = 10/group) were maintained on a diet of chow supplemented with 0.2% (w/w) cholesterol for 6 weeks. On days 3 and 5 of the last week of dietary supplementation the animals received two infusions of saline, rHDL (8 mg/kg apoA-I) or ETC-642 (30 mg/kg peptide). Blood was collected at sacrifice and HDL were isolated and their composition determined as described in "Materials and Methods". Results are expressed as mean ± SEM.

## Discussion

The development of apoA-I mimetic peptides has provided much therapeutic promise for the treatment of inflammatory and cardiovascular diseases. ETC-642 is a 22 amino acid peptide that mimics the structure of the amphipathic α-helices of apoA-I when combined with lipids. We therefore compared its ability to inhibit chronic vascular inflammation in cholesterol-fed rabbits, with that of rHDL (containing apoA-I and PLPC). We find, for the first time, that the mimetic peptide ETC-642 inhibits adhesion molecule expression in this model. Furthermore, HDL isolated from rabbits injected with ETC-642 displays more potent anti-inflammatory properties than HDL isolated from rabbits injected with saline or rHDL.

Increased expression of adhesion molecules VCAM-1 and ICAM-1, represent the earliest vasculature alteration following the initiation of an atherogenic diet [17,18]. Using a cholesterol-fed rabbit model, vascular inflammation develops gradually via a process that is somewhat comparable to early human lesion development [17]. In this model we have demonstrated that infusions of rHDL were able to significantly attenuate cholesterol-induced aortic expression of VCAM-1 and ICAM-1. These findings support the numerous *in vitro *studies that demonstrate the anti-inflammatory properties of rHDL and *in vivo *studies showing that rHDL and apoA-I inhibit acute vascular inflammation [3,6,13,19]. Furthermore, this study shows for the first time that the mimetic peptide ETC-642 can inhibit adhesion molecule expression in a model of chronic vascular inflammation. Our findings are also consistent with previous studies that have used ETC-642 and shown that it inhibits the development of similar inflammatory pathologies such as acute inflammation in the carotid artery in the rabbit peri-arterial collar model [16], cholesterol-induced aortic valve stenosis [20] and atherosclerosis in apoE^-/- ^mice [21].

The beneficial cardio-protective properties of HDL have been attributed to their ability to efflux cholesterol from cells, including endothelial cells [22,23]. Several studies have demonstrated that apoA-I mimetic peptides are also able to stimulate cholesterol efflux [13,24]. For example, infusions of ETC-642 transiently elevate cholesterol levels in rats and humans [20,25], indicating enhanced mobilization of cell cholesterol into the plasma. Enhanced cholesterol efflux may provide a mechanism for the anti-inflammatory effects of ETC-642, as has been demonstrated by other mimetic peptides [13]. In the current study, however, we found that ETC-642 had no effect on plasma total cholesterol or HDL cholesterol concentrations at the time of sacrifice. The reason for this is likely to be that the final ETC-642 injection was 48 hours prior to sacrifice. As the cholesterol elevating properties of ETC-642 occur within 45 minutes after infusion [20], the lack of change at 48 h post-infusion is therefore to be expected [16]. This may also explain why HDL composition did not change following rHDL and ETC-642 infusions and confirms our recent work, which showed that injection of lipid free apoA-I and rHDL increased HDL phospholipid, unesterified cholesterol and cholesterol ester levels at 5 minutes, but not at 6 h after infusion [26].

Despite the lack of apparent compositional change in rabbit HDL between treatment groups, the HDL from rabbits infused with ETC-642 appeared to exert more potent anti-inflammatory effects *ex vivo *in HCAECs than the HDL from rabbits injected with saline or rHDL. Recent proteomic studies have identified multiple proteins that co-isolate with human HDL [27,28], including for example antioxidants, protease inhibitors and growth factors. These HDL-associated proteins are likely to impart some of the beneficial physiological effects of HDL [27]. Changes in the presence of these HDL-associated proteins are also likely to alter HDL function. It is possible that whilst the ETC-642 infusions did not significantly alter the relative amounts of phospholipids, unesterified cholesterol, esterified cholesterol or triglycerides, it may have changed the protein composition of the rabbit HDL in a way that increased their anti-inflammatory properties. Interestingly, the HDL from ETC-642 injected mice were also able to reduce the protein levels of the active subunit of NF-κB, p65. This reduction in p65 provides a possible mechanism for the reductions in both VCAM-1 and ICAM-1 as they are both regulated via activation of the NF-κB pathway [29,30]. This is also consistent with a number of studies showing that HDL and rHDL reduce NF-κB activation and the expression of p65 [6,13,31].

The current study demonstrated that infusions of rHDL and ETC-642 inhibit the initial inflammatory response. This suggests that rHDL and ETC-642 may also attenuate the next stages of atherosclerotic lesion development. In support of this, previous work by our group and others has demonstrated that rHDL reduces lesion size and macrophage content in rabbits and mice [19,32,33]. Furthermore, adenoviral gene transfer of ETC-642 has been shown to decrease atherosclerosis in apoE^-/- ^mice [21].

## Conclusions

Using a cholesterol-fed rabbit model of chronic vascular inflammation we have demonstrated that mimetic peptide ETC-642 inhibits endothelial expression of adhesion molecules, markers of the earliest stages of atherosclerosis. These studies provide additional support for the therapeutic efficacy of mimetic peptides in the treatment of inflammation and cardiovascular disease.

## Methods

### Isolation of rabbit HDL and apolipoprotein A-I

Rabbit HDL were isolated from rabbit plasma (100 ml) by sequential ultracentrifugation in the 1.063 < d < 1.21 g/ml density range [34] and dialyzed against endotoxin-free phosphate buffered saline (PBS). Human HDL were isolated from pooled samples of autologously donated human plasma (Gribbles Pathology, South Australia) by sequential ultracentrifugation in the same density range. The human HDL were delipidated and apoA-I isolated by anion chromatography [34].

### Preparation of rHDL containing apoA-I and PLPC

Discoidal rHDL containing apoA-I complexed to 1-palmitoyl-2-linoleoyl phosphatidylcholine (PLPC) (Avanti Polar Lipids, Alabaster, AL, USA) (initial PLPC/apoA-I molar ratio 100:1). were prepared using the cholate dialysis method [35]. The resulting rHDL were dialysed extensively against endotoxin-free PBS (pH 7.4) before use.

### Preparation of ETC-642

The ETC-642 was provided as a lyophilized solid by Esperion Therapeutics, Pfizer (Groton, MI, USA). The complex consists of ESP-2418, an amphipathic peptide containing 22 L-amino acid residues: P-V-L-D-L-F-R-E-L-L-N-E-L-L-E-A-L-K-Q-K-L-K with a molecular weight of 2623 daltons that is complexed to sphingomyelin and 1,2-dipalmitoyl-*sn-*glycero-3-phosphocholine (DPPC) (peptide/sphingomyelin/DPPC molar ratio 1/3.75/3.75. The lyophilized solid was rehydrated with 50 ml of a sterile bicarbonate saline solution (Esperion Therapeutics, Pfizer, Groton, CT, USA) and gently swirled to mix. Three cycles of warming at 50°C and cooling to room temperature were performed to ensure complete reconstitution. The final protein concentration of ETC-642 was 10 mg/ml and the peptide was stored at 4°C until use.

### Animal Experiments

The Sydney South West Area Health Service Animal Welfare Committee approved all animal procedures. Thirty male NZW rabbits weighing approximately 3 kg were obtained from the Institute of Medical and Veterinary Sciences (Adelaide, South Australia). Following a seven-day acclimatisation period, baseline plasma cholesterol and HDL levels were determined prior to placing the animals on a diet of normal chow supplemented with 0.2% (w/w) cholesterol for 6 weeks. Five weeks after commencement of the diet, the rabbits were randomised into three treatment groups (n = 10/group). A blood sample (3 mL) was collected from each animal at this time point. Plasma cholesterol levels were then determined to confirm that there were no significant differences between treatment groups. The animals then received two intravenous infusions of saline (control group), rHDL (8 mg/kg apoA-I) or ETC-642 (30 mg/kg peptide) on the third and fifth days of the final (sixth) week of dietary intervention and were sacrificed at the end of the sixth week of high cholesterol feeding. The animals were euthanased with an intravenous overdose of sodium pentobarbital (100 mg/kg). Plasma was collected via cardiac puncture. The thoracic aortae were then removed, flushed with chilled PBS before placement in cold 4% (v/v) paraformaldehyde overnight. The aortae were then transferred to 70% (v/v) ethanol and stored at 4°C, then paraffin embedded for histological analysis.

### Immunohistochemistry

Sections (5 mm) of the paraffin-embedded vessels were cut, dewaxed, and rehydrated. Mouse anti-rabbit VCAM-1 and ICAM-1 antibodies (gifts from Dr M Cybulsky, University of Toronto) were used to assess endothelial expression of adhesion molecules in the thoracic aorta. For the assessment of macrophages, thoracic aortic sections were stained for RAM11 (1:100; Dako). Thoracic aortic sections (10 sections/rabbit) were analysed using ImagePro Plus 4.5 (Media Cybernetics, Silver Spring, MD) as described [3,19]. Image analysis results are expressed as image units.

### Plasma Analyses and HDL composition

Total cholesterol concentrations were determined on rabbit plasma and isolated rabbit HDL enzymatically using commercially available kits (Roche Diagnostics). HDL cholesterol concentrations were determined on rabbit plasma by enzymatic assay following precipitation of apolipoprotein B containing lipoproteins with polyethylene glycol [36]. Phospholipid, unesterified cholesterol, cholesterol ester and triglyceride concentrations were assessed in rabbit HDL also using commercially available kits (Wako Pure Chemicals).

### Endothelial cell culture

Human coronary artery endothelial cells (HCAECs) were grown in MesoEndo cell culture medium (Cell Applications Inc., CA, USA) and seeded at 1 × 10^6 ^cells/well in 12-well plates. Cells were then treated for 24 h with PBS, rHDL (1 mg/ml final apoA-I concentration) or isolated rabbit HDL (1 mg/ml final protein concentration). Following this cells were washed 1× with PBS and then stimulated with tumor necrosis factor-α (TNF-α) (1 ng/ml). After 5 h the cells were harvested and stored at -80°C until required.

### Real-time PCR

Total RNA was extracted using the Trizol method, quantitated and normalised to 100 ng/uL using SYBR Green II assay (Invitrogen). RNA was then reverse transcribed in triplicate to cDNA using iSCRIPT (BioRad). Real-time PCR was performed in triplicate using iQSYBR Green Supermix (BioRad) and an iCycler iQ Real-time thermocycler (BioRad). The threshold cycle was calculated using iCycler iQ Real-time PCR detection system software version 3.0A (BioRad). Relative changes in mRNA expression between treatments were determined using the cT method. Results were normalised using GAPDH.

### Statistical Analyses

All results are expressed as mean ± SEM. Statistical comparisons were made by two-tailed Student's t-tests and one way ANOVA with Bonferroni's test *post hoc *using GraphPad Prism Version 4.0 (San Diego, CA). A value of p < 0.05 was considered statistically significant.

## Abbreviations

(apoA-I): Apolipoprotein A-I; (apoE): Apolipoprotein E, (DPPC): 1,2-dipalmitoyl-*sn*-glycero-3-phosphocholine, (GAPDH): Glyceraldehyde 3-phosphate dehydrogenase, (HDL): High-density lipoproteins, (HCAECs): Human coronary artery endothelial cells, (ICAM-1): Intracellular cell adhesion molecule-1, (mRNA): Messenger ribonucleic acid, NZW rabbits: New Zealand White rabbits, (NF-κB): Nuclear factor kappa-light-chain-enhancer of activated B cells, (p65): p65 subunit of NF-κB, (PLPC): 1-palmitoyl-2-linoleoyl phosphatidylcholine, (PBS): Phosphate buffered saline, (rHDL): Reconstituted HDL, (TNF-α): Tumour necrosis factor-α; (VCAM-1): Vascular cell adhesion molecule-1.

## Competing interests

The authors declare that they have no competing interests.

## Authors' contributions

BAD-Performed *in vivo *and *in vitro *studies as well as the histology. Also assisted in the preparation of the manuscript. LZV-Performed quantitative PCR experiments. JTMT-Assisted in *in vitro *studies and quantitative PCR experiments. SB-Performed analysis of histological sections. KAR-Conceived the project and experimental design and assisted in the preparation of the manuscript. PJB-Conceived the project and assisted in the preparation of the manuscript. CAB-Assisted with the experiments and prepared the manuscript. All authors have read and approved the manuscript.
